# Analytical approaches to examine gamma-aminobutyric acid and glutamate vesicular co-packaging

**DOI:** 10.3389/fnsyn.2022.1076616

**Published:** 2023-01-04

**Authors:** SeulAh Kim, Bernardo L. Sabatini

**Affiliations:** Department of Neurobiology and Harvard Medical School, Howard Hughes Medical Institute, Boston, MA, United States

**Keywords:** co-transmission, glutamate, GABA, electrophysiology, statistical analysis

## Abstract

Multi-transmitter neurons, i.e., those that release more than one type of neurotransmitter, have been found in many organisms and brain areas. Given the peculiar biology of these cells, as well as the potential for diverse effects of each of the transmitters released, new tools, and approaches are necessary to parse the mechanisms and functions of synaptic co-transmission. Recently, we and others have studied neurons that project to the lateral habenula and release both gamma-aminobutyric acid (GABA) and glutamate, in some cases by packaging both transmitters in the same synaptic vesicles. Here, we discuss the main challenges with current electrophysiological approaches to studying the mechanisms of glutamate/GABA co-release, a novel statistical analysis that can identify co-packaging of neurotransmitters versus release from separate vesicle, and the implications of glutamate/GABA co-release for synapse function and plasticity.

## 1. Introduction

Glutamate and gamma-aminobutyric acid (GABA) are fast-acting neurotransmitters used by nervous systems throughout most of phylogeny. In the mammalian brain, by acting on distinct sets of ionotropic receptors, these transmitters generally produce opposing effects on a target neuron, with glutamate typically exciting and GABA inhibiting the post-synaptic neuron. Interestingly, there are neurons that have the pre-synaptic machinery necessary to release both glutamate and GABA and indeed do use both transmitters to signal to their post-synaptic partners (Root et al., 2014; Wallace et al., 2017). These are among the many classes of neurons that release multiple transmitters, including those that release multiple fast-acting neurotransmitters that signal through ionotropic receptors (e.g., GABA and acetylcholine), a single fast-acting neurotransmitter and a second small molecule that signals through G-protein coupled receptors (e.g., GABA and dopamine), or a classical small-molecule transmitter and a neuropeptide (e.g., GABA and somatostatin) (Nusbaum et al., 2001; Vaaga et al., 2014; Tritsch et al., 2016). The prevalence and diversity of multi-transmitters synapses suggests that they are fundamental to normal brain function; however, our understanding of the functions afforded by different combinations and mechanisms of transmitter co-release is lagging due to limited tools to study synapses at which multi transmitters are released.

Where and how each transmitter is synthesized, transported, packaged, released, and recycled are tightly linked to the actions of co-transmitters. Neuropeptide and small classic molecule transmitters are usually packaged in separate vesicles (small clear synaptic vesicles vs. large dense core vesicles). Therefore, even when released from single cells, each typically has different capacities for sustained release, requirements for intracellular Ca^2+^ for vesicle mobilization, and post-synaptic targets (Nusbaum et al., 2001; Hnasko and Edwards, 2012). In contrast, neurotransmission involving the release of multiple small molecules from individual cells is more heterogeneous. In some cases, fast-acting neurotransmitters are co-released from individual vesicles as result of the action of a shared transporter that recognizes more than one substrate (Wojcik et al., 2006; Melani and Tritsch, 2022) or co-localization of multiple transporters on single vesicles (Hnasko et al., 2010). In other cases, each small molecule transmitter is packaged in its own class of vesicle, which can then be trafficked to and released from segregated pre-synaptic active zones (Root et al., 2014; Zhang et al., 2015; Granger et al., 2020). Therefore, diverse mechanisms of co-release of multiple fast-acting neurotransmitters make elucidating mechanisms and actions of these synapses more challenging.

Co-release is defined as the liberation of two or more classical neurotransmitters *via* vesicle fusion in response to a physiological stimulus, and thus refers to a property of a pre-synaptic neuron. Depending on the mechanisms of co-release, the release of each transmitter may have different frequency dependence and post-synaptic targets (Nusbaum et al., 2001). Co-transmission refers to the release and detection of multiple co-released molecules by a target neuron, and thus is a property of a pair of pre- and post-synaptic neurons. Hence, for both transmitters to contribute to synaptic transmission at a single synapse, the target cell needs to express both receptors for both molecules. The numerous possible modes of co-release and the potential for independent regulation of post-synaptic receptors for each transmitter, allows great functional diversity at co-transmitting synapses.

Neurons that release both glutamate and GABA are rare, and, in the mammalian brain, tend to project to one brain region, the lateral habenula (LHb) (Hu et al., 2020; Xu et al., 2022). LHb is involved in cognition and mood disorders *via* modulation of downstream serotonergic and dopaminergic centers (Hikosaka, 2010; Hu et al., 2020). Interestingly, pre-synaptic terminals of projections from several brain regions co-release glutamate/GABA or converge glutamatergic and GABAergic inputs onto individual LHb cells (Hu et al., 2020; Xu et al., 2022). Nevertheless, the function of such co-transmission remains mysterious. The mechanisms of co-release appear to differ depending on the origin of these axons (Shabel et al., 2014; Wallace et al., 2017; Root et al., 2018, 2014; Kim et al., 2022), suggesting that it likely has important functional implications for synaptic integration and function of LHb.

**FIGURE 1:**
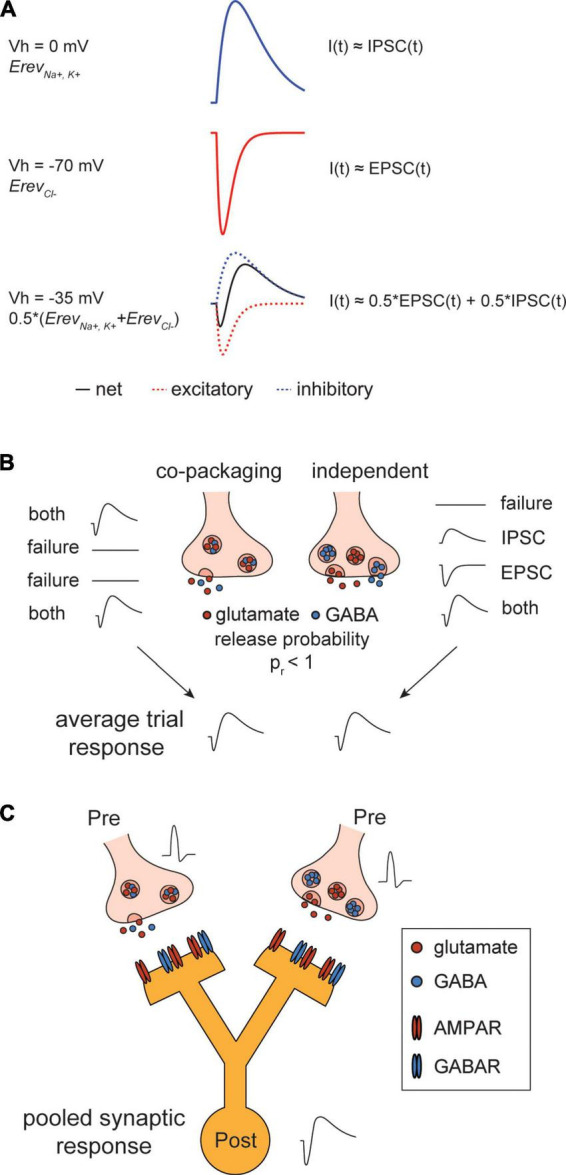
Post-synaptic currents arising from glutamate/gamma-aminobutyric acid (GABA) co-release. **(A)** Composite signal of glutamate/GABA co-release is biphasic. Top, simulated post-synaptic current (PSC) at reversal potential of AMPA-type glutamate receptor (AMPAR) (Vh = 0 mV). At this membrane potential, net current is inhibitory PSC (IPSC) dominant. Middle, simulated PSC at reversal potential of GABAR (V_h_ = –70 mV). At this membrane potential, net current is excitatory PSC (EPSC) dominant. Bottom, simulated PSC at intermediate potential of the two receptors (V_h_ = –35 mV). The net current is biphasic with reduced amplitudes of inward and outward currents. **(B)** Stochastic vesicle release causes PSC distributions to diverge between “co-packaging” and “independent” models of glutamate/GABA co-release but their normalized average trial responses are identical. Left, two types of PSCs arise from vesicles that co-package glutamate and GABA in individual vesicles/both and failure modes. Right, four types of PSCs arise from vesicles that independently package glutamate and GABA/ IPSC, EPSC, both and failure modes. Bottom, averaging across trials under the two modes of co-release result in a biphasic PSC. **(C)** Schematic showing the actions of co-activated pre-synaptic boutons that differentially co-release glutamate/GABA converging onto a same post-synaptic cell. Pooled synaptic response detected at the soma of a downstream neuron reflects a biphasic response although activating the “independent” site may transmit only an EPSC.

Recently, we (Kim et al., 2022) demonstrated that terminals of entopeduncular projections in LHb co-package glutamate and GABA inside the same synaptic vesicles. Using repeated optogenetic stimulations of an individual co-releasing pre-synaptic bouton under conditions of stochastic vesicle release, the statistical properties of mixed glutamatergic/GABAergic post-synaptic currents (PSCs) could be analyzed. Analysis of trial-to-trial variability of these biphasic PSCs supported a model in which the two neurotransmitters are in the same rather than separate synaptic vesicles. In this review we discuss the challenges of studying co-releasing synapses with electrophysiological methods and describe methods that are useful in examining the mechanisms and consequences of co-release in individual synapses. We focus on methods that can distinguish if two transmitters are packaged in the same synapse (the “co-packaging” model) vs. released independently from separate vesicle pools located in the same pre-synaptic terminal (the “independent” model) (Figure 1). We also discuss implications of glutamate/GABA co-release and co-transmission in context of computation and plasticity.

## 2. Challenges to studying co-releasing synapses

To determine the organization of putative co-releasing pre-synaptic terminals and distinguish co-packaging vs. independent release of multiple transmitters, several obstacles must be solved. First, one needs to know which pre-synaptic terminals to study, a step that can be difficult in the dense neuropil of the mammalian brain which typically contains axons and synapses from many molecularly and anatomically distinct neurons. Second, one needs to establish if an individual synapse contains vesicles for multiple transmitters and, subsequently, if each vesicle contains only one or multiple neurotransmitters. Third, techniques, typically optical or electrophysiological, are necessary to trigger and detect the precisely timed activation of a single terminal and vesicle. Fourth, for the study of co-transmission, one needs to be able to independently detect the action of each transmitter, even if the small quantal signals produced by each transmitter generate signals of opposite polarity. Lastly, measurements and models of the signals produced by co-transmitter synapses are needed to be able to quantitatively describe and distinguish the modes of multi-transmitter release. With these requirements in mind, below we describe molecular and electrophysiological approaches that have been used to examine if two transmitters are co-packaged in individual vesicles and how each satisfies the criteria listed above.

## 3. Proteomic approaches for examining neurotransmitter co-packaging

Immunohistochemical labeling of endogenous proteins associated with the release of each neurotransmitter (i.e., those necessary for the synthesis, packaging, or detection of these molecules) allows comparison of the spatial distributions of synaptic machinery associated with one or the other neurotransmitter. The immunolabeled tissue can then be imaged at the ultrastructural level with electron microscopy or super-resolution light-microscopy to examine the proximity of proteins associated with handling of each neurotransmitter (Shabel et al., 2014; Root et al., 2018, 2014). However, this approach has several potential limitations (Gonda, 1998), including the need to establish the specificity of each antibody for its intended epitope in the tissue of interest and under the particular analysis modality used, which can be particularly challenging for electron microscopy. Similarly, the penetrance of the antibody labeling–i.e., what fraction of epitopes for a specific antibody are recognized and detected–may be low due to molecular crowding, low affinity of the antibody, or non-ideal conditions for antibody labeling. Low penetrance might preclude detection and accurate quantification of co-labeling, even if proteins are always found in proximity, such as on the same vesicle. On the other hand, the combined length of the primary and secondary antibodies (∼30 nm) is on the same order as the size of the synaptic vesicle, making it hard to conclude true co-localization at the vesicular level (Takamori et al., 2006; Reth, 2013). Newer approaches, such as epitope-preserving expansion microscopy in which antibodies are introduced after tissue expansion (Chen et al., 2015; M’Saad et al., 2022) may be of particular benefit to determining the distribution of proteins across individual synaptic vesicles.

Alternatively, proteomic and metabolomic analysis can be performed on purified synaptic vesicles to examine putative co-packaging vesicles. Various combinations of proteomic techniques have been implemented such as co-immunoprecipitation, immunogold staining with electron microscopy, or mass spectrometry (Shabel et al., 2014; Root et al., 2018). However, cell-fractionation and affinity purification often do not preserve information on the cellular origin of the individual synaptic vesicles which provides genetic and circuit context necessary for functional perturbations and studies. Moreover, studies of pooled vesicles yield population level results and, thus, are typically not suitable for revealing the neurotransmitter profile of a single genetically and molecularly defined neural or synaptic vesicle population. Recently, novel methods have been developed to address some of these issues, including profiling neurotransmitter content from synaptic vesicles captured directly from brain tissues in a cell-type-specific manner (Chantranupong et al., 2020). However, these also examine bulk properties of vesicles isolated from a cell and cannot make conclusion about the contents of individual vesicles. Nevertheless, they may be useful to examine co-packaging by, for example, immuno-isolating vesicles based on expression of a vesicular GABA transporter and then probing them for glutamate content.

## 4. Electrophysiological methods

The gold standard for analysis of synaptic physiology remains whole-cell recordings of synaptic currents, which can be used to examine the downstream actions of neurotransmitter release. For the study of co-releasing synapses, because the technique relies on recording of currents evoked in a post-synaptic cell, it is restricted to the study of co-transmitting synapses. Below we consider fast synaptic transmission-based readouts which provide high fidelity temporal information about neurotransmitter release onset and the correlations between currents evoked by multi-transmitters.

### 4.1. General principles

Action-potential evoked co-release of neurotransmitters leads to activation of multiple post-synaptic ionotropic receptors (if both are expressed) and subsequent current flow through these channels. In mammalian neurons, glutamate activates fast excitatory ionotropic receptors, including AMPA-type glutamate receptors (AMPARs) which mediate most of the inward current underlying excitatory PSCs (EPSCs). Conversely, GABA activates fast inhibitory ionotropic receptor GABA_A/C_ receptors (GABARs), resulting in outward inhibitory PSCs (IPSCs). When both currents are generated near simultaneously, they overlap, resulting in a net composite, biphasic signal with reduced inward and outward components (Figure 1A). These currents may be measured separately [by holding membrane potential to reversal potentials of each receptor, AMPARs (∼0 mV) and GABARs (∼−60 mV) or through sequential pharmacological isolation of each signal] or simultaneously by holding at a potential intermediate (e.g.,−35 mV) to the reversal potentials. The latter approach is necessary for the analyses of trial-by-trial correlations in the appearance, timing, and amplitude of the currents.

### 4.2. Bulk measurements of co-release

When multiple pre-synaptic terminals are stimulated at the same time, either with an extracellular electrode or with optogenetics, many synapses will activate near synchronously, leading to a bulk PSC. In this case, differential kinetics related to channel opening and closing can be used to identify neurotransmitter content from the mixed PSC measurements. In the case of glutamate/GABA co-release, AMPARs have significantly faster kinetics than GABARs and the respective currents are further distinguished by their signs [i.e., inward (−) and outward (+)].

However, other currents evoked by the simultaneous action of multiple transmitters may not be readily separated if they have the same polarity and similar kinetics. Typically, mixed currents mediated by co-release are sequentially isolated by applying specific pharmacological blockers and observing kinetics changes in the remaining current (Jonas et al., 1998; Li et al., 2004; Lamotte d’Incamps et al., 2017). These approaches establish that co-transmission occurs, but because both transmitters are not measured at the same time, correlations of and interactions between their release cannot be evaluated.

Examining the short-term plasticity at multi-transmitter synapses is often informative about the mechanisms of release (Jonas et al., 1998; Silm et al., 2019). Transmitters sharing a synaptic vesicle are expected to exhibit the same pre-synaptic release properties such as Ca^2+^ dependence and short-term processes underlying synaptic strength changes (Zucker and Regehr, 2002). Paired pulse ratio (PPR), which measures the level of depletion of readily releasable vesicles pool by the first evoked response, can be compared between transmitters to test if the two neurotransmitter release pathways diverge. However, one needs to be careful to characterize and correct for potential post-synaptic contributions to PPR, such as receptor desensitization or saturation. Furthermore, such analyses must typically be performed on each class of PSC in isolation; hence, they face similar issues as above of not being able to measure these properties simultaneously for the two transmitters. As bulk PSC measurements destroy statistical information regarding variability across synapses and synaptic vesicles, they are not able to draw conclusion about pre-synaptic properties or kinetics of receptors attributed to a single terminal or single vesicle.

### 4.3. Single vesicle measurements of co-release

If both receptors are expressed in a post-synaptic cell, the spontaneous quantal release of a co-packaging vesicle will evoke a PSC with contributions of channels opened by multiple transmitters. To measure mixed miniature spontaneous PSCs, a post-synaptic target is typically whole-cell voltage clamped in the presence of tetrodotoxin to block sodium-based action potentials. Mixed miniature PSCs have been observed in neurons that receive synapses that co-release glycine and GABA (Jonas et al., 1998; Awatramani et al., 2005), glutamate and Ach (Li et al., 2004; Borodinsky and Spitzer, 2007), and glutamate and GABA (Shabel et al., 2014; Zimmermann et al., 2015). Although this approach measures single vesicle responses, one generally does not know the identity of the pre-synaptic terminal or cell that released the vesicle. Therefore, the scarcity of these events renders the statistical analysis of their features difficult and, given their unknown source, cannot be used to determine the properties of multiple release events from a single terminal or to make comparisons across terminals.

A more direct approach to test co-release at putative synaptic sites is to evoke single vesicle release by stimulating an axon at a “minimal” intensity at which release becomes stochastic. Mixed kinetics quantal events associated with defined set of electrically stimulated synapses have been demonstrated in several co-releasing sites (Gillespie et al., 2005; Lamotte d’Incamps et al., 2017). Rigorous statistical analysis is required to conclude that the minimally evoked synaptic currents arise from a unitary connection or single synapse (Stevens and Wang, 1995). Electrical minimal stimulation generally cannot differentiate the signals produced by vesicles released from multiple synaptic terminals within the same axon.

Substitution of strontium for calcium is often used to desynchronize release of vesicles from an electrically- or optogenetically-stimulated axons, allowing the study of single vesicle evoked responses (Oliet et al., 1996; Bekkers and Clements, 1999; Lamotte d’Incamps et al., 2017). Strontium enhances delayed release and affects short-term plasticity at various synapses (Xu-Friedman and Regehr, 1999, 2000), spreading out individual release events across hundreds of milliseconds. It is difficult to sample from a single synapse repeatedly using this approach unless it is coupled with a minimal stimulation; thus, strontium is a useful tool to combine with specific stimulation strategies to generate dataset capturing single trial vesicle dynamics.

### 4.4. Analysis of co-release measurements

Single vesicle PSCs (spontaneous miniature and evoked) have been typically studied using receptor antagonists to determine whether two transmitters are released together. Upon blocking currents mediated by one transmitter, a change in the kinetics of remaining evoked PSCs suggest that unitary responses carry components arising from two transmitters (Jonas et al., 1998; Gillespie et al., 2005). However, such analysis is unable to resolve trial-by-trial vesicle dynamics from a single release site and differentiate heterogeneous synapses or vesicles.

Another approach to analyze single vesicle PSCs is to model the composite signal as a sum of individual transmitter mediated currents using template matching. Each current can be modeled roughly with two exponential functions that govern its rising and falling phases (Clements and Bekkers, 1997). In total, this approach requires fitting six unknown parameters (i.e., on/off kinetics and amplitude for each of two currents). Furthermore, one needs to estimate these six parameters trial-by-trial. In our experience (and as expected for a relatively unconstrained problem), this template matching approach often fails to identify a unique solution in analysis of glutamate/GABA mediated biphasic currents. For the case of currents with the same polarity, template matching would be more problematic (and likely impossible if the kinetics of the receptors are similar).

Whether the two neurotransmitters are in the same or segregated pools of vesicles cannot be determined by averaging responses across multiple stimulation of individual synapses because the mean responses will appear identical (Figure 1B). Moreover, pooled responses from co-activation of multiple synapses likely occlude the heterogeneity of vesicles, which is an important feature of the “independent” model (Figure 1C). However, the two models can be differentiated by analyzing the trial-to-trial variance of PSCs elicited from a single synapse stimulated many times. In this case, it is important to be able to measure both currents at the same time to examine their co-variance and to know that only one synapse has been stimulated per trial.

Recent advances in optogenetic control of neural activity and optical techniques permit manipulating individual synapses to release one vesicle at a time (Kim et al., 2022). Glutamatergic and GABAergic currents monitored using whole-cell recordings in voltage-clamp mode are distinguished by their signs [i.e., inward (−) and outward (+)] when the cell is held at a potential intermediate to the GABA and glutamate receptor reversal potentials. The single vesicle dynamics from the same synapse is then analyzed to describe how well the statistics support each candidate model of co-release.

To properly analyze evoked, single-vesicle responses, two problems must be solved. First, one must be able to distinguish stimulation trials in which vesicle fusion occurred (i.e., successes) from those in which it did not (i.e., failures trials). Second, for success trials, one must be able to infer when vesicle release (or PSCs) occurred. Both problems are difficult to solve because of the small and variable size of the currents as well as the variability in vesicle fusion and PSC timing. These are made more difficult when trying to judge the timing of both inward and outward currents, particularly those, like glutamatergic and GABAergic currents, that have opposing sign and overlap temporally.

## 5. Statistical analysis of single vesicle dynamics obtained from the glutamate/gamma-aminobutyric acid co-releasing terminals

We developed a statistical approach to study biphasic synaptic currents arising from the actions of opposing transmitters. Simulations of post-synaptic currents reveal a close link between heterogeneity and variability of PSCs and how the two transmitters are packaged into vesicles. Under a stochastic vesicle release condition, packaging of the two transmitters into separate vesicles that are released independently is predicted to result in PSCs that vary more than those resulting from vesicles containing both transmitters (Figure 1B).

**FIGURE 2:**
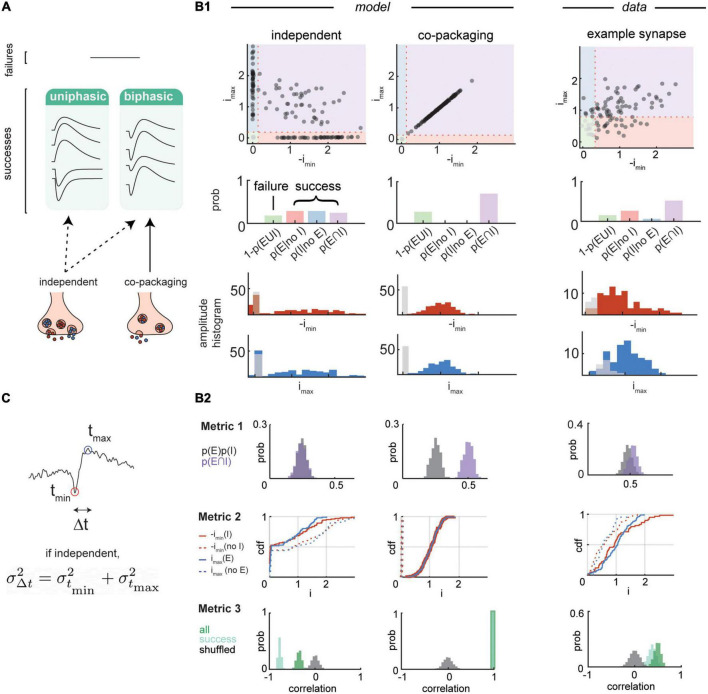
Single vesicle post-synaptic current dynamics analysis. **(A)** Classifications of simulated post-synaptic currents (PSCs) under a low noise condition. First, individual trials of PSCs can be sorted into failure and success modes. Next, individual success trials of PSCs can be categorized to either biphasic or uniphasic modes. Neurotransmitter co-release by segregated vesicles predicts heterogeneity of the PSCs, containing both uniphasic and biphasic events. Co-release by co-packaging vesicles predicts presence of only biphasic success events. **(B1,B2)** Three statistical metrics for distinguishing co-release models and their application to experimental data analysis. Data adapted from Kim et al. (2022). **(B1)** Top, scatterplots of the maximum and minimum amplitudes of simulated PSCs (200 trials) from independent (left) and co-packaging (middle) release models compared to that of an example synapse (98 trials) (right). Amplitudes are normalized to the average maximum (*y*-axis) and minimum (*x*-axis) amplitudes of success trials. Middle, bar graphs of probabilities of trials with failures of both PSCs (green), success of excitatory PSC (EPSC) only (pink), success of inhibitory PSC (IPSC) only (blue), and success of both PSCs (purple). Bottom, histograms of the maximum (blue) and minimum (red) amplitudes with success of release and failures of release (gray). **(B2)** Analysis of the three metrics are compared between simulated dataset from independent (left) and co-packaging (middle) release models and an example synapse dataset (right). Top, Metric 1 determines if glutamate and GABA currents occur in individual trials more frequently than that expected by chance. Bootstrapped probability of presence of both EPSCs and IPSCs (purple) and multiples of individual EPSC and IPSC probabilities (gray) are shown. Middle, Metric 2 determines whether conditions of presence of absence of one current influence the distribution of the other current. Cumulative distribution functions of IPSC amplitude (i_max_, blue) conditional on the presence [i_max_ (E), solid] or the absence [i_max_ (no E), dashed] of EPSC. Similar analyses were performed on the EPSC amplitude [i_min_, red; i_min_ (I), solid; i_min_ (no I), dashed]. Bottom, Metric 3 determines if glutamate and GABA current amplitudes are correlated trial-by-trial. Bootstrapped correlation of a pair of EPSC and IPSC amplitudes for all trials (dark green), success trials (light green), and all trials with pairing order shuffled (gray). **(C)** Temporal variance of biphasic PSCs. Minimum (red dot) and maximum (blue dot) amplitude peaks were extracted from the analysis time window and their timings are indicated by t_min_ and t_max_, respectively. Δt is the time difference between t_min_ and t_max_ within a trial.

Classification of trials based on PSC shapes is useful for analyzing the mechanisms of co-release of multiple transmitters. Under a low noise condition, biphasic events are expected to occur in both co-packaging and independent release conditions, whereas uniphasic events should occur only in the independent release case (Figure 2A; Kim et al., 2022). However, experimental data of individual synapses contain noises from various sources, contributing to a phenotype intermediate to the two co-release models and necessitating more sophisticated features (Figure 2B). Quantitative analysis of statistical features of the PSC frequencies and amplitudes can determine if PSCs collected from a repetitive activation of a single synapse support co-packaging (“co-package model”) or independent packaging of the two transmitters (“independent model”). Luckily, much of the statistical analysis can be performed by examination of minimum and maximum peak amplitudes of PSCs in each trial, even if some of these PSCs represent noise (i.e., failures of synaptic release). In cases in which the amplitudes of failures and successes are not well separated, Gaussian model fitting of individual cell’s noise can be used as a threshold for judgment of presence or lack of signal.

We used three statistical analyses of these peaks that differentiate co-packaging and independent release models and differentially require accurate identification of “success” and “failure” trials, which can often be difficult given the small sizes of currents resulting from the release of individual vesicles (Figure 2B).

- Metric 1 quantifies how well a set of PSCs obeys the statistical independence of release by comparing the probability of both glutamate- and GABA-currents occurring in single trials compared to the probability of the two events occurring by chance if each release occurred independently. Only in the case of glutamate and GABA co-release from the same vesicle, the two currents will occur more frequently in each trial than the multiplies of probabilities of each current. This method relies on correct judgment of presence or absence of EPSCs and IPSCs in single trials.
- Metric 2 examines how presence or absence of one current influences the distribution of amplitude of the other current. This method involves grouping trials based on one current, then comparing distributions of the other current given the sorting scheme. If co-packaging occurs, the grouping method will influence the distributions; however, if the two currents are independent, then the distributions will be not impacted by how we group the events.
- Metric 3 tests whether the glutamate- and GABA-mediated currents co-vary in their amplitude sizes across trials. The expected correlation coefficient of the two currents is positive in the co-packing model (as result of sharing of vesicle-level variation in its content size and jitter) and negative in the independent model (reflecting subtraction of opposite polarity PSCs).

We find that many glutamate/GABA co-releasing synapses are in support of the co-packaging model (Figure 2B) that are not explained by known artifacts impacting the analysis results, including experimental noise, biological noise, and activation of multiple release sites. In fact, these artifacts will tend to make co-packaging sites appear more independent according to the three metrics.

## 6. Secondary statistical metrics

In addition to analyzing variance in the amplitude, variance in the timing of maximum (t_max_) and minimum (t_min_) peaks in the biphasic trials can serve as a secondary metric for distinguishing between the two release models (Figure 2C). The release of a neurotransmitter containing vesicle occurs with some jitter (Katz and Miledi, 1965a,b; Diamond and Jahr, 1995; Chen and Regehr, 1999), such that the timing of the peak of a well-isolated GABA or AMPA receptor currents should show variance relative to the stimulus onset. The variances of separation in time of the two current peaks will be:


$$\sigma_{△\,\text{t}}^{2}=\sigma_{{\text{t}}_{\text{min}}}^{2}+\sigma_{{\text{t}}_{\text{max}}}^{2}-2\,C\,o\,v\,\left({\text{t}}_{\text{min}},{\text{t}}_{\text{max}}\right),$$

where the Cov(t_min_,t_max_) indicates the covariance between the two peak timings. If GABA and glutamate are released independently, covariance between the timing of the maximum and minimum currents should equal to zero such that variance of timing difference will equal to sum of independent variances $\sigma_{{\text{t}}_{\text{max}}}^{2}$ and $\sigma_{{\text{t}}_{\text{min}}}^{2}$. However, if both transmitters are released together, then variances of the timing of the peaks arise from a common source–the jitter in timing of release of one vesicle. In the extreme of no recording noise and perfect determination of the timing of the peaks, this will lead to $\sigma_{△\,\text{t}}^{2}=0$. In practice, the current noise in the recording will obscure the exact timing of each peak and add variance. Nevertheless, observing that $\sigma_{△\,\text{t}}^{2}<\sigma_{{\text{t}}_{\text{min}}}^{2}+\sigma_{{\text{t}}_{\text{max}}}^{2}$ indicates a positive covariance between the two currents, which violates the independent release of glutamate and GABA.

An alternative examination of PSC variance over time also makes different predictions for co-packaged versus independent release without the requirement of extracting the timing of current peaks. Using the same logic as above, the time point at which mean trace crosses zero is expected to have minimum variance in the co-packaging model but large variance in the independent release model. This difference may be challenging to detect in practice when analyzing experimental data as it requires alignment of PSC traces across trials to eliminate jitter caused by variable timing of vesicle release. In theory, this can be done with template matching (Rey et al., 2015) but, in practice, can lead to a circular argument because, if biphasic currents are individually aligned using information about the zero crossing time, then the variance of the currents will reach a minimum at the time of the mean trace zero crossing by construction.

## 7. Alternative methods for measuring glutamate/gamma-aminobutyric acid co-release

Non-electrophysiology approaches exploiting the wonderful new tool kit of neurotransmitter and neuromodulator sensitive fluorescent proteins can potentially be used to simultaneously measure the release of multiple transmitters. This includes examining glutamate and GABA release *via* multiplex imaging using iGluSnFR and iGABASnFR. These sensors were derived from periplasmic binding protein (PBP) attached to fluorescent proteins such that ligand binding alters fluorescence. They can traffick into or near synaptic clefts to directly measure the timing and magnitude of neurotransmitter transients (Helassa et al., 2018; Marvin et al., 2019, 2013; Sabatini and Tian, 2020). With a 2-photon microscope, this approach can be used to resolve spatiotemporal concentration changes occurring at an individual synapse (Dürst and Oertner, 2022), which is not feasible with the whole-cell recordings. Complications of measuring both transmitters using fluorescent sensors include compensating for differential sensitivity, binding kinetics, and bleaching rates, which may need to be addressed during the *post-hoc* analysis of these signals. Furthermore, although there has been tremendous progress in glutamate sensors (Aggarwal et al., 2022) and they have the speed and sensitivity to detect single release events in “real-world” experimental conditions, the same does not seem to be true for GABA sensors.

Yet another approach is to pair imaging with electrophysiology measurements. For example, whole-cell recordings can be performed at 0 mV to measure GABAR currents elicited while glutamate release is monitored *via* iGluSnFR. This approach has several potential benefits: (1) electrophysiology signal quality improves by measuring only GABA currents (in fact, glutamate receptors can be blocked pharmacologically), (2) we gain site specificity of transmitter release, and (3) we gain access to direct measurement of transmitter release, even potentially when it is sub-threshold for channel opening.

## 8. Functional implications of glutamate/gamma-aminobutyric acid co-release

Co-release of two fast acting, opposing neurotransmitters may have functional implications for the maturation of synapses and the computations underlying perception and cognition. Several regions including auditory brain stem and hippocampus seem to use co-release of glutamate/GABA for synaptic refinement during brain development. In the lateral superior olive of auditory circuit, co-release of a third small molecule, glutamate, by inhibitory GABA/glycinergic co-releasing inputs from medial nucleus of the trapezoid body supports activity-dependent synapse refinement that is important for sound localization (Gillespie et al., 2005). In hippocampus, GABA is transiently released by glutamatergic granule cells of dentate gyrus to provide post-synaptic GABA-mediated depolarization needed for N-methyl-D-aspartate (NMDA) receptor activation (Leinekugel et al., 1997; Gutiérrez et al., 2003; Gutiérrez, 2005). Hence, glutamate and GABA co-release drives the refinement and fine-tuning of microcircuits.

How computations afforded by different modes of glutamate/GABA co-release relate to function in an adult brain remains more elusive. One example is supramammillary projection neurons that co-release glutamate and GABA onto Golgi cells and interneurons in the dentate gyrus (Pedersen et al., 2017; Hashimotodani et al., 2018). Optogenetic activation of this pathway partially promotes wakefulness, but it is unclear how the opposing effects of the neurotransmitters affect cell firing and contribute to arousal. Another example is multiple of glutamate/GABA co-releasing synapses found in LHb (Hu et al., 2020; Xu et al., 2022). The computations and functions carried out by these are not well understood.

The functional impact of different modes of co-release on synaptic integration is unclear. We speculate a potential role of co-packaging glutamate and GABA from a computational perspective: a post-synaptic cell, while being agnostic to pre-synaptic modulation and differentiation, can assign a signed and graded weight to each synapse by adjusting the numbers of GABA and glutamate receptors in the post-synaptic terminal. This provides a PSC with a guaranteed sign and with consistent ratio of glutamate and GABA currents as it does not depend on the stochastic and independent release of GABA vs. glutamate containing vesicles. This could simplify plasticity rules for the downstream neurons in determining what information to extract and transform from upstream neurons.

Moreover, the mode at which glutamate/GABA co-release occurs has implications for differential regulatory mechanisms used to balance excitatory and inhibitory transmissions in a circuit, potentially providing mechanic explanations for physiology underlying disrupted brain functions. For example, studies in rodents reveal that shifted glutamate/GABA co-transmission appear in altered LHb circuits in mood disorders (Li et al., 2011; Shabel et al., 2014; Meye et al., 2016; Hu et al., 2020). Yet, we do not know whether the computational function of glutamate/GABA co-release is compromised in these conditions. If glutamate and GABA are co-packaged in the same vesicles, homeostatic processes that adjust vesicular content may be impaired, whereas if they are released separately, one or several of the regulatory pathways could be impacted. Interestingly, several neuromodulators, such as serotonin and adenosine, modulate the probability of release of glutamate/GABA co-packaging vesicles in LHb (Kim et al., 2022), suggesting potential role of neuromodulation in shifting the contribution of co-releasing synapses to computation in the LHb.

When glutamate/GABA co-packaging vesicles are released during behavior and what they signal remain to be determined. Although it is likely that these vesicles are released by each action potential produced in a neuron–in fact, they may constitute all of the vesicles of such neurons–the possibility remains that they are a subset of vesicles released only at certain synapses or in specific conditions. Therefore, investigating how the unique computations carried out by the co-packaging vesicles support cognitive functions mediated by specialized brain region will be important areas of future research.

## Author contributions

Both authors wrote the manuscript, contributed to the article, and approved the submitted version.
